# AI in Surgical Curriculum Design and Unintended Outcomes for Technical Competencies in Simulation Training

**DOI:** 10.1001/jamanetworkopen.2023.34658

**Published:** 2023-09-19

**Authors:** Ali M. Fazlollahi, Recai Yilmaz, Alexander Winkler-Schwartz, Nykan Mirchi, Nicole Ledwos, Mohamad Bakhaidar, Ahmad Alsayegh, Rolando F. Del Maestro

**Affiliations:** 1 Neurosurgical Simulation and Artificial Intelligence Learning Centre, Department of Neurology and Neurosurgery, Montreal Neurological Institute and Hospital, McGill University, Montreal, Quebec, Canada; 2 Faculty of Medicine and Health Sciences, McGill University, Montreal, Quebec, Canada; 3 Division of Neurosurgery, Department of Surgery, Faculty of Medicine, King Abdulaziz University, Jeddah, Saudi Arabia

## Abstract

**Importance:**

To better elucidate the role of artificial intelligence (AI) in surgical skills training requires investigations in the potential existence of a hidden curriculum.

**Objective:**

To assess the pedagogical value of AI-selected technical competencies and their extended effects in surgical simulation training.

**Design, Setting, and Participants:**

This cohort study was a follow-up of a randomized clinical trial conducted at the Neurosurgical Simulation and Artificial Intelligence Learning Centre at the Montreal Neurological Institute, McGill University, Montreal, Canada. Surgical performance metrics of medical students exposed to an AI-enhanced training curriculum were compared with a control group of participants who received no feedback and with expert benchmarks. Cross-sectional data were collected from January to April 2021 from medical students and from March 2015 to May 2016 from experts. This follow-up secondary analysis was conducted from June to September 2022. Participants included medical students (undergraduate year 0-2) in the intervention cohorts and neurosurgeons to establish expertise benchmarks.

**Exposure:**

Performance assessment and personalized feedback by an intelligent tutor on 4 AI-selected learning objectives during simulation training.

**Main Outcomes and Measures:**

Outcomes of interest were unintended performance outcomes, measured by significant within-participant difference from baseline in 270 performance metrics in the intervention cohort that was not observed in the control cohort.

**Results:**

A total of 46 medical students (median [range] age, 22 [18-27] years; 27 [59%] women) and 14 surgeons (median [range] age, 45 [35-59] years; 14 [100%] men) were included in this study, and no participant was lost to follow-up. Feedback on 4 AI-selected technical competencies was associated with additional performance change in 32 metrics over the entire procedure and 20 metrics during tumor removal that was not observed in the control group. Participants exposed to the AI-enhanced curriculum demonstrated significant improvement in safety metrics, such as reducing the rate of healthy tissue removal (mean difference, −7.05 × 10^−5^ [95% CI, −1.09 × 10^−4^ to −3.14 × 10^−5^] mm^3^ per 20 ms; *P* < .001) and maintaining a focused bimanual control of the operative field (mean difference in maximum instrument divergence, −4.99 [95% CI, −8.48 to −1.49] mm, *P* = .006) compared with the control group. However, negative unintended effects were also observed. These included a significantly lower velocity and acceleration in the dominant hand (velocity: mean difference, −0.13 [95% CI, −0.17 to −0.09] mm per 20 ms; *P* < .001; acceleration: mean difference, −2.25 × 10^−2^ [95% CI, −3.20 × 10^−2^ to −1.31 × 10^−2^] mm per 20 ms^2^; *P* < .001) and a significant reduction in the rate of tumor removal (mean difference, −4.85 × 10^−5^ [95% CI, −7.22 × 10^−5^ to −2.48 × 10^−5^] mm^3^ per 20 ms; *P* < .001) compared with control. These unintended outcomes diverged students’ movement and efficiency performance metrics away from the expertise benchmarks.

**Conclusions and Relevance:**

In this cohort study of medical students, an AI-enhanced curriculum for bimanual surgical skills resulted in unintended changes that improved performance in safety but negatively affected some efficiency metrics. Incorporating AI in course design requires ongoing assessment to maintain transparency and foster evidence-based learning objectives.

## Key Points

**Question:**

Is use of artificial intelligence (AI) in a simulated surgical skills curriculum associated with unintended performance outcomes?

**Findings:**

This cohort study of 46 medical students and 14 experts found that the AI-enhanced simulation curriculum demonstrated significant unintended changes in 52 performance metrics outside the curriculum that were not observed in the control cohort. These unintended changes included significantly improved procedural safety (eg, healthy tissue damage) but significantly worsened movement (eg, dominant hand velocity) and efficiency (eg, rate of tumor removal) metrics.

**Meaning:**

This cohort study found that use of AI in designing a simulated surgical skills curriculum was associated with unintended learning outcomes, with both positive and negative consequences for learner competency, suggesting that intervention from human experts may be required to optimize educational goals.

## Teaser

This cohort study is a secondary analysis of a randomized clinical trial and assesses the pedagogical value of technical competencies selected by artificial intelligence (AI) and their extended effects in surgical simulation training.

## Introduction

In surgical education, technical competency is one of the key factors of efficacy in the curriculum, and it is an independent factor that is directly associated with postoperative patient outcomes.^1,2^ By gathering large amounts of data from surgical simulation training, machine learning and deep learning algorithms can be used to design intelligent tutoring systems to deliver a curriculum enhanced with artificial intelligence (AI).^3,4,5^ An intelligent tutoring system is a pedagogical tool powered by an AI model that can provide learners with tailored performance assessment and feedback.^6^ Developing such systems requires large amounts of data from users with varying levels of skill on standardized procedures that can be obtained from virtual reality simulators.^4,7^

Simulation is an important component of competency-based medical education.^8^ Technical skills acquired in simulation training have been demonstrated to improve operating room performance and lead to better health outcomes for patients.^9,10^ Curriculum competencies are determined by a committee of subject matter experts, academics, educators, and researchers through a transparent and evidence-based approach.^11^ With the vast amount of performance and neurophysiologic data collected during virtual reality simulation and the ability to apply AI technology, AI tutors using machine learning algorithms may be useful tools to provide novel educational insights and define quantifiable competency metrics.^3,12^ It is therefore warranted to apply the same standards of transparency and academic rigor in evaluating the competencies selected and taught by AI systems.

In our previous work,^4^ an AI-enhanced curriculum was developed to teach a neurosurgical technique in virtual reality simulation to medical students. The learning objectives of this curriculum were chosen by an algorithm, a support vector machine, that analyzed a pool of 270 metrics and selected 4 that were most significantly associated with expertise.^3,4^ These included 2 metrics related to safety (bleeding rate and maximum force applied by the nondominant hand) and 2 metrics related to movement (maximum acceleration of the nondominant hand and the instrument tip separation distance).^6^ Feedback on these metrics was delivered by the Virtual Operative Assistant (VOA), an intelligent tutoring system that evaluates learners’ competency level in safety and movement, and provides personalized post-hoc audiovisual feedback.^4^ In a randomized clinical trial, the effectiveness of this AI-enhanced curriculum was compared with remote instruction by experts on the technical skills of medical students in simulation training.^13^ The findings of this study suggested that feedback on AI-selected learning objectives could have an effect beyond the performance criteria taught by the AI tutor.^14^ However, the scale and mechanism of this extended effect has not been previously reported in simulation training and it remains unclear whether such effects have a beneficial or detrimental effect on students’ skill.

To assess the pedagogical value of AI-selected competencies, we explored their extended associations with other performance criteria and investigated how this changed students’ competency compared with the level of skilled surgeons. To do so, we used a cohort study design by following up medical student’s exposure in the VOA group and comparing their performance outcomes with a control cohort and a skilled cohort. We hypothesized that medical students exposed to the VOA will demonstrate significant changes on several performance criteria related to the AI-selected competencies, and that the observed extended effects are partially responsible for achieving expert performance benchmarks.

## Methods

### Study Participants

We conducted a planned secondary analysis using retrospective data from 2 previously conducted studies, a cohort study of 14 experts and a randomized clinical trial involving 46 medical students. In the first study, skilled consultants performed a simulated neurosurgical procedure with no feedback to establish expert benchmarks.^3^ In the second study, medical students were randomized into 2 groups and learned to perform the same task with or without instructions from the AI-enhanced curriculum.^14^ Both studies were approved by the McGill University Health Centre Research Ethics Board, Neurosciences-Psychiatry, and all participants signed an approved informed consent form before trial participation. This article follows the Strengthening the Reporting of Observational Studies in Epidemiology (STROBE) reporting guideline and the Best Practices for Machine Learning to Assess Surgical Expertise.^15^

### Study Procedure and Simulation

All participant performed 5 simulated neurosurgical tumor resection procedures within a fixed time and either received an intervention (VOA group) or no intervention (control and skilled groups) after the completion of each attempt.^3,14^ Participants received standardized verbal and written instructions on the simulated task, the goals of the procedure, and the instruments used. Additionally, they performed an orientation module to navigate the 3-dimensional virtual reality space and test each instrument’s functions. The intervention for the VOA group involved receiving post-hoc audiovisual instructions on 4 learning objectives based on the learners’ lacking competency. This curriculum follows a stepwise competency assessment in which learners must first achieve expert classification for safety metrics in step 1 before moving to step 2 to learn instrument movement metrics.^14^ Procedures were performed on the NeuroVR (CAE Healthcare) virtual reality simulator that records the state of 54 variables in the operation, such as tumor size or the 3-dimensional position of each instrument, at a 50 Hz rate (*t* = 20 ms).^16,17^ The raw data were collected and used to generate assessment metrics.

### Performance Metric Extraction and Expertise Benchmarks

Raw data from the initial and final attempts were used to extract 270 assessment metrics for each procedure. These metrics were selected based on their ability to differentiate experts from novices and are equally divided into 3 groups depending on the state of the operation: during tumor resection, while suctioning blood, or over the entire scenario.^3^ Because of large variability in the duration and amount of blood loss, 90 metrics from the suctioning blood state were excluded from analysis. Data from the skilled group were used to determine expertise benchmarks for each metric by measuring the mean score with 1 SD following previously published protocols.^18,19^ The benchmark provides a reference to evaluate whether the extended changes in medical student performance metrics have a positive or negative impact on their competency. All metrics with a significant within-participant difference from baseline in the VOA group that did not significantly change in the control group were the primary outcome measures.

### Statistical Analysis

Collected data were examined for normality with the Shapiro-Wilk test, and Wilcoxon rank test was used when *t* test assumptions were not satisfied. Outliers were identified using boxplot analysis and Levene test was used to assess variance. In procedures in which there was no blood loss, metrics related to bleeding were not applicable. Any metric in any group with more than half the data missing was excluded in the statistical analysis. Within-participant comparison of medical students’ performance in 180 metrics in both the VOA and the control group was performed by 2-sided paired samples *t* test (α = .05) to identify metrics that demonstrated a significant change between baseline (first attempt) and after the intervention (fifth attempt). Between-participants comparison of performance metrics at baseline and after the intervention was conducted with independent samples *t* tests. *P* values were not adjusted for multiple testing to avoid being overly conservative and subsequently missing important treatment outcomes.^20,21^ Data were analyzed using MATLAB release 2022a (The MathWorks) and SPSS Statistics version 28 (IBM) statistical software from June to September 2022.

## Results

All 46 medical students (median [range] age, 22 [18-27] years; 27 [59%] women) and 14 surgeons (median [range] age, 45 [35-59] years; 14 [100%] men) completed the trial, and no one was lost to follow-up. Medical students were in the preclinical period of their studies (19 students [41%] in premedicine program; 16 students [35%] in first year of medical school; and 11 students [24%] in second year of medical school) and had minimal surgical experience.^14^ The skilled group had a median (range) of 12.5 (1-25) years of practice experience as neurosurgical staff, with most participants (9 participants [64%]) primarily involved in cranial surgery.^3^ Further participant demographic information is presented in the Table.

**Table.** Demographic Characteristics of Participants

Characteristic	Medical students, No. (%)		Staff neurosurgeons, skilled group (n = 14), No. (%)
	Control group (n = 23)	AI instruction group (n = 23)	
Age, median (range), y	22 (18-26)	21 (19-27)	45 (35-59)
Gender			
Men	9 (39)	10 (43)	14 (100)
Women	14 (61)	13 (57)	0
Undergraduate medical training level			
Preparatory	9 (39)	10 (43)	NA
First year	8 (35)	8 (35)	NA
Second year	6 (26)	5 (22)	NA
Institution			
McGill University	14 (61)	8 (35)	14 (100)
University of Montreal	3 (13)	7 (30)	0
University of Laval	6 (26)	7 (30)	0
University of Sherbrooke	0	1 (5)	0
Dominant hand			
Right	23 (100)	21 (91)	14 (100)
Left	0	2 (9)	0
Interest in pursuing surgery, mean (SD)^a^	3.7 (1.0)	3.9 (1.1)	NA
Total time in practice, median (range), y	NA	NA	12.5 (1-25)
Neurosurgical subspecialty			
Spine	NA	NA	5 (36)
Oncology and epilepsy	NA	NA	4 (29)
Skull base	NA	NA	2 (14)
Pediatrics	NA	NA	2 (14)
Cerebrovascular	NA	NA	1 (7)

Abbreviations: AI, artificial intelligence; NA, not applicable.

^a^ Range, 1 to 5, with higher scores indicating greater motivation to pursue a surgical specialty in postgraduate training.

**Figure 1.**
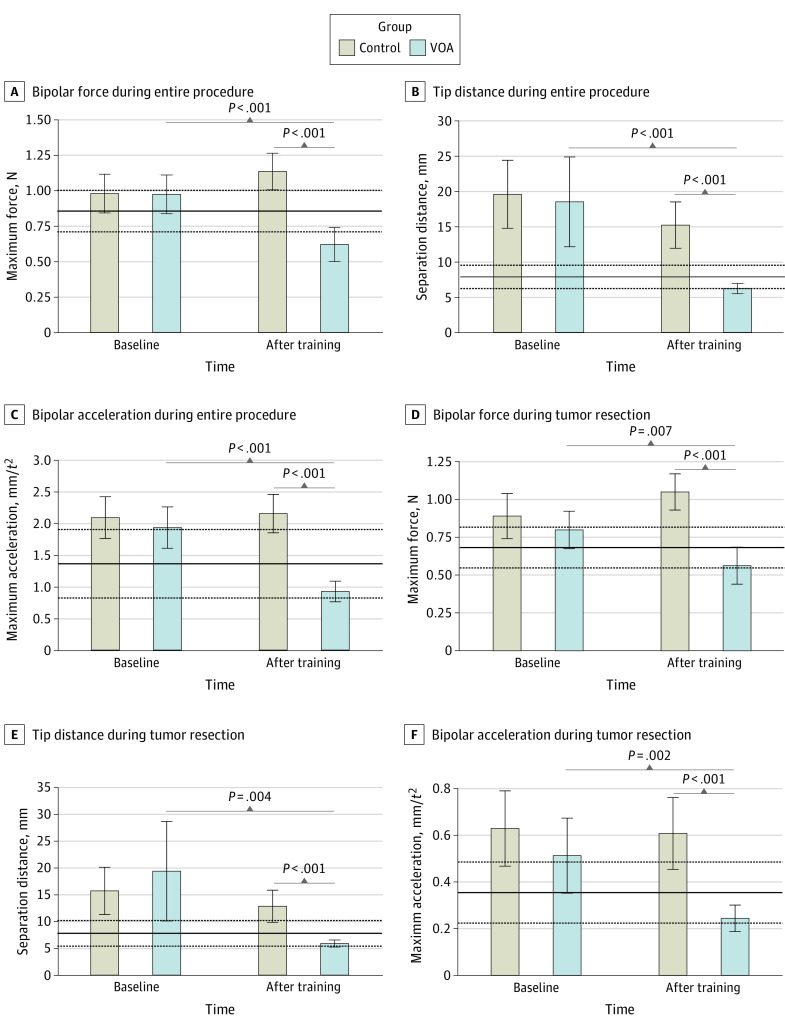
Performance in the Learning Objectives of the Virtual Operative Assistant (VOA) Curriculum The skilled group’s mean and SD in each metric are represented by the solid and dashed lines, respectively. Because the variables are recorded at a frequency of 50 Hz by the simulator, each unit of time (t) is equal to 20 ms. Error bars indicate 95% CIs.

Participants in the VOA group responded successfully to performance feedback and achieved expert benchmarks in the curriculum’s learning objectives (Figure 1). At the end of the AI-enhanced curriculum, learners in this group demonstrated significant performance change in 42 metrics during the entire procedure and 20 metrics during tumor resection. Within these metrics, control participants also demonstrated significant performance change in 10 metrics during the entire procedure and no metrics during tumor resection. Therefore, instruction on 4 AI-selected learning objectives was associated with a significant extended performance change in 32 metrics over the entire procedure and 20 metrics during tumor resection that was not observed in the control group. Complete analyses of all affected metrics in the 2 conditions are provided in eTable 1 and eTable 2 in Supplement 1.

**Figure 2.**
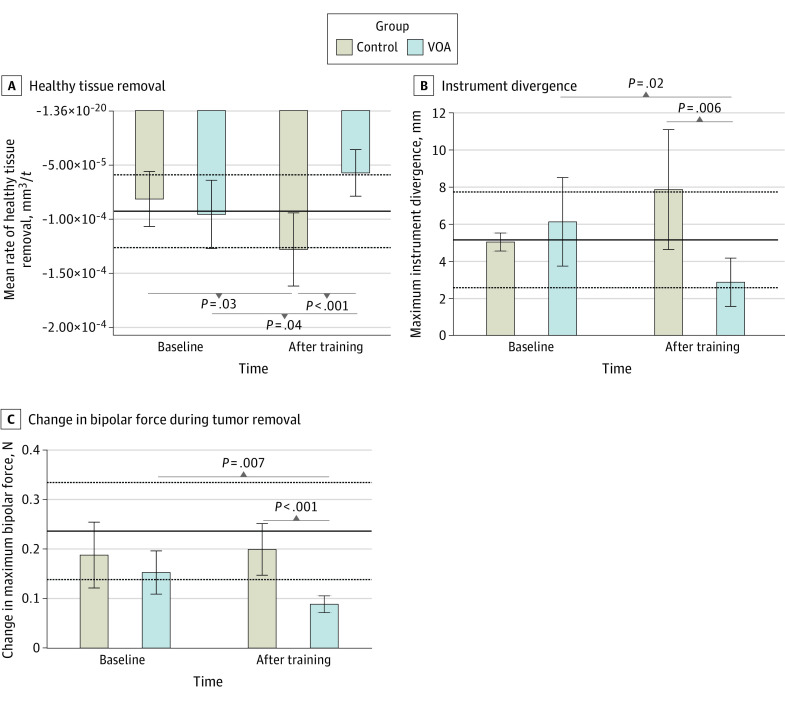
Extended Associations of the Virtual Operative Assistant (VOA) Curriculum With Safety Competencies The skilled group’s mean and SD in each metric are represented by the solid and dashed lines, respectively. Because the variables are recorded at a frequency of 50 Hz by the simulator, each unit of time (t) is equal to 20 ms. Error bars indicate 95% CIs.

Damage to healthy brain tissue is an important safety metric in neurosurgical tumor resection surgery.^22^ In this study, participants in the VOA group demonstrated a significant reduction in rate of healthy tissue removal (mean difference, 3.82 × 10^−5^ [95% CI, −7.51 × 10^−5^ to −1.21 × 10^−6^] mm^3^/t; *P* = .04). By the end of the training, VOA participants’ tumor resection technique resulted in significantly less damage to surrounding brain compared with the control group (mean difference, −7.04 × 10^−5^ [95% CI, −1.10 × 10^−4^ to −3.08 × 10^−5^] mm^3^/t; *P* < .001). Participants in the control group demonstrated a significant increase in their rate of healthy tissue damage (mean difference, 4.64 × 10^−5^ [95% CI, 5.43 × 10^−6^ to 8.74 × 10^−5^] mm^3^/t, *P* = .03). Participants receiving AI feedback achieved the expert benchmark (mean [SD], 9.26 × 10^−5^ [3.35 × 10^−5^] mm^3^/t) in this safety metric (Figure 2A).

Two other important metrics of safety are maintaining a focused bimanual control of the operative field and applying steady force with the bipolar forceps in the nondominant hand. We explored these qualities by looking at instrument divergence and rate of change in the forces applied by the nondominant hand during tumor resection, respectively (Figure 2B-C). The maximum divergence of instruments was significantly reduced at the end of training in the AI group (mean difference, −3.24 [95% CI, −5.88 to −0.61] mm; *P* = .02) whereas it did not significantly change in the control group (mean difference, 2.82 [95% CI, −0.35 to 6.00] mm; *P* = .08). After training, the VOA group achieved a significantly lower instrument divergence compared with the control group (mean difference, −4.99 [95% CI, −8.48 to −1.49] mm; *P* = .006) and achieved the expert benchmarks (mean [SD], 5.14 [2.57] mm) (Figure 2B).

Compared with baseline, the change in maximum bipolar force during tumor resection was significantly reduced in the VOA group (mean difference −6.40 × 10^−2^ [95% CI, −1.09 × 10^−2^ to −1.86 × 10^−2^] N/t; *P* = .007) and not in the control group by the end of training (mean difference, 1.14 × 10^−2^ [95% CI, −7.08 × 10^−2^ to 9.36 × 10^−2^] N/t, *P* = .78). After training, the VOA group had a significantly lower force change compared with the control group (mean difference, −0.11 [95% CI, −0.17 to −0.06] N/t; *P* < .001); however, they fell below the expert benchmark (mean [SD], 0.24 [0.10] N/t) (Figure 2C).

**Figure 3.**
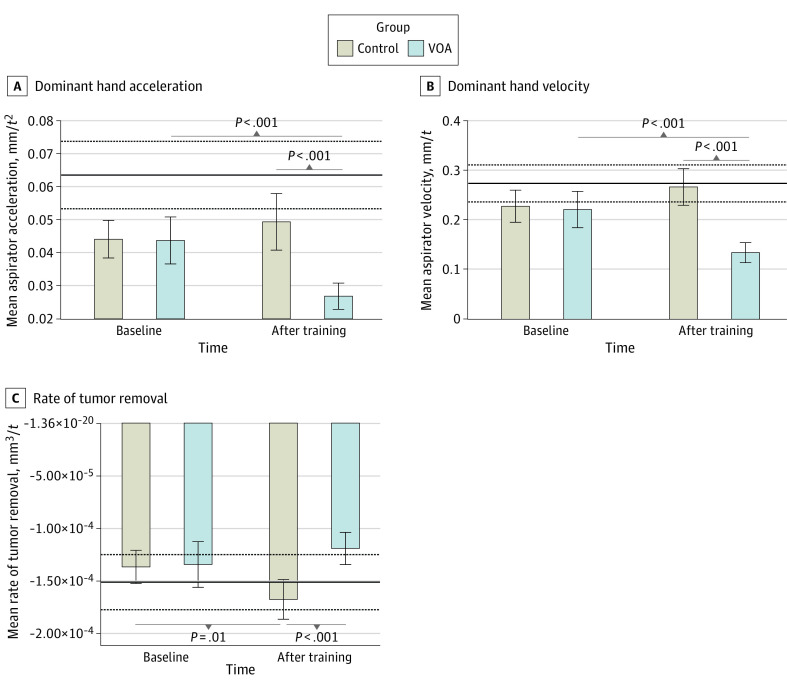
Extended Associations of the Virtual Operative Assistant (VOA) Curriculum With Movement and Efficiency Metrics The skilled group’s mean and SD in each metric are represented by the solid and dashed lines, respectively. Because the variables are recorded at a frequency of 50 Hz by the simulator, each unit of time (t) is equal to 20 ms. Error bars indicate 95% CIs.

VOA participants also demonstrated significant changes in movement metrics of their dominant hand. Notably, by the end of the AI curriculum, students performed with a significantly lower velocity (mean difference, −0.13 [95% CI, −0.17 to −0.09] mm/t, *P* < .001) and lower acceleration (mean difference, −2.25 × 10^−2^ [95% CI, −3.20 × 10^−2^ to −1.31 × 10^−2^] mm/t^2^; *P* < .001) in their dominant hand compared with the control group. This unintended effect persisted over the whole procedure and during tumor resection, and it diverged performance away from the lower threshold of the expertise benchmark in both metrics (dominant hand velocity: mean [SD], 0.27 [0.04] mm/t; dominant hand acceleration: 6.35 × 10^−2^ [1.02 × 10^−2^] mm/t^2^) (Figure 3A-B).

Finally, we looked at the rate of tumor resection over the entire procedure to appreciate the efficiency of performance. In this metric, although the control group significantly increased their rate (mean difference, 3.10 × 10^−5^ [95% CI, 7.01 × 10^−6^ to 5.49 × 10^−5^] mm^3^/t, *P* = .01), the AI curriculum was not associated with a significant increase. After the intervention, the VOA group had a significantly lower rate of tumor resection compared with no intervention (mean difference, −4.85 × 10^−5^ [95% CI, −7.22 × 10^−5^ to −2.48 × 10^−5^] mm^3^/t; *P* < .001). This diverged performance in the VOA group further below the expertise benchmark in this metric (mean [SD], 1.51 × 10^−4^ [2.62 × 10^−5^] mm^3^/t) (Figure 3C).

## Discussion

This cohort study is the first study to demonstrate unintended surgical skill acquisition with both positive and negative consequences following an AI-enhanced curriculum, to our knowledge. In the competency-based framework of postgraduate medical training, AI provides a tool to identify and teach quantifiable metrics of expertise. Harnessing this power in neurosurgical simulation led our team to design and test the first curriculum with AI-selected competencies. The previous randomized trial involving medical students compared the efficacy of AI tutoring with remote expert instruction using performance outcomes that included only the 4 metrics taught by the VOA.^14^ This study is focused on only the cohort who were exposed to the AI-enhanced curriculum and explores 270 novel aspects of their performance to investigate the extent of this mode of instruction. This study also builds on the previous report that identified metrics of expertise by comparing medical students’ performance outcome with computed expertise benchmarks. Similar to other clinical learning environments that result in formal and informal learning,^23,24^ this novel curriculum demonstrated both intentional and unintentional learning outcomes.

Metrics affected by this AI-enhanced curriculum fall into 1 of 3 categories. The first group includes the intrinsic metrics taught by the intelligent tutoring system. Because the feedback provided by the tutor was focused on these preselected learning objectives, observing the expected outcome provides evidence for feedback efficacy. The second group involves implicit metrics, such as instrument divergence or changes in bipolar force, that despite receiving no direct feedback demonstrated significant change by virtue of their close association with the intrinsic learning objectives. The last group are extrinsic metrics, such as the rate of healthy tissue removal, that have a more complex relationship with the intended learning objectives that cannot be easily implied.

Among extrinsic metrics, changes in dominant hand movement were an interesting observation because the students were instructed by the tutor to monitor the acceleration of their nondominant hand and to keep their instruments close together. Nondominant hand training was previously demonstrated to be an effective mode of surgical skill acquisition because of its close association with expertise.^25,26,27^ Our results demonstrate an interesting intermanual skill transfer whereby training the nondominant hand was associated with movement changes of the dominant hand. The underlying mechanism behind this observation is not clearly understood; however, functional asymmetry in the brain may offer an explanation. A study using functional near-infrared spectroscopy in individuals learning a manual skill with their nondominant hand showed difference in participants’ cortical activation patterns with nondominant hand training, notably bilateral premotor cortex activation, compared with those who trained with their dominant hand.^28^ This suggests that perhaps the awareness required to control nondominant hand movements results in a change of movements of the dominant hand due to functional dominance and network asymmetry between hemispheres.^28,29^ It is notable that all 4 AI-selected learning objectives required expert nondominant hand performance; 2 were direct measurements from the bipolar forceps, while the other 2 required bimanual control.

It is possible that the modality of feedback presentation contributes to the extended effects observed in this study. As part of the VOA feedback, participants were exposed to four 60-second videos of both competent and novice performance covering each learning objective. Although instructions in each video were specific to 1 metric, participants could potentially gain further information to improve their performance by seeing the expert demonstrations. However, this fails to explain the divergence from benchmarks in some efficiency and movement metrics.

The extended effects of this AI curriculum show both productive and counterproductive changes to skill acquisition. Although VOA’s unintentional learning outcomes resulted in students scoring within expert safety benchmarks for this procedure, it diverged some efficiency metrics away from the expert’s benchmarks. In general, VOA participants demonstrated a safer approach with more focused and steady control of the instruments that also resulted in less healthy tissue damage. However, they became significantly slower in movements of their dominant hand and were less efficient in removing the tumor. Such unintended effects must be considered carefully and require a cost-benefit evaluation by experts to determine the acceptability of an intelligent tutoring system. Furthermore, given the extent of unintended outcomes observed in this study, future studies may benefit from measuring the slope of each metric’s learning curve to investigate the learning rate elicited by an AI-enhanced curriculum using extrinsic performance outcomes.

One method to evaluate the gains and losses of skill due to unintended effects is to refer to participants’ previously reported expert-rated Objective Structured Assessment of Technical Skills. These results demonstrated that the VOA group achieved a significantly higher overall performance score and a higher economy of movement compared with the control group.^14^ This is interesting because despite the observed loss of efficiency in rate of tumor removal and slow bimanual movements, blinded expert evaluators still rated the VOA participants as having a better economy of movement than the control group. This discrepancy demonstrates 1 limitation of a highly granular assessment of expertise based on individual metrics because performance in each domain is composed of the interaction among its multiple constituting metrics. Perhaps with increased capacity to collect surgical performance data, future studies can use advanced AI algorithms to redefine subjective areas of performance (eg, economy of movement or flow) as functions consisting of the interaction between multiple metrics and project performance on the Objective Structured Assessment of Technical Skills scale. This would not only provide instructors with a more meaningful understanding of their student’s performance but can focus their assessment and feedback on specific measurable criteria.

### Future Directions

With advancements in live intraoperative data acquisition for quality improvement, AI systems are believed to play a key role in mitigating adverse events and suggesting instructions in the operating room.^5,30,31,32,33^ Rolling out intelligent systems for patient care or formal postgraduate training should occur in phases and requires the same rigor of scientific practice required for pharmacologic therapies or other medical devices. For our study, this would involve investigating the outcomes of an AI-augmented curriculum designed in collaboration with the neurosurgical training program to offer a hybrid of expert instruction with the state-of-the-art Intelligent Continuous Expertise Monitoring System training in a simulated operative environment with realistic tissues.^34,35,36^ AI applications will continue to expand in medical education and hold promise to enhance health care by providing timely data-driven analytics.^37^ However, we need to be cognizant of the potential for their unintended effects and promote transparency to maximize beneficial outcomes for learners and patients.

### Limitations

This study has some limitations. One limitation is the number of surgeons in the skilled group and their range of experience in neuro-oncological surgery. However, most participants in the skilled group primarily practiced cranial neurosurgery, and subpial resection is a fundamental technique that is mastered throughout postgraduate training.^38^ Furthermore, previously validated models trained on this sample could not only distinguish expertise but project residents’ postgraduate training year in neurosurgery.^3,5,39^ Although best practice is to compare trainee performance with quantifiable criterion standards of experts, training medical students to achieve benchmarks closer to their competency level, for example that of a junior resident, may prove more practical.^40,41,42^ The results of a previous study demonstrated that VOA participants could achieve a greater overall expertise score than a junior resident but not that of a senior resident or a staff surgeon.^5,14^ Whether this method of training results in more realistic performance goals for junior learners or it delays the mastery learning curve warrants longitudinal studies that investigate long term effects of exposure to AI-enhanced curricula.

Students in the VOA group were limited to only post-hoc performance feedback provided by the AI tutor, which lacks important contextual and directional components. For example, unidirectional instructions to reduce force or acceleration at the end of each attempt are likely to result in cumulative rather than time-specific behavioral changes.^43,44^ Furthermore, pausing the operation interrupts negative momentum and provides an opportunity for immediate reflection on poor performance that has greater learning value and can prevent intraoperative errors.^45^ To address this, we developed an Intelligent Continuous Expertise Monitoring System capable of error projection and real-time feedback delivery.^5^ This system’s efficacy is being evaluated and compared with live instruction from expert tutors in a randomized clinical trial.^46,47^ This trial will be able to determine the value of pausing live performance to deliver real-time instructions and investigate whether similar unintended effects are observed with continuous performance feedback.

## Conclusion

This cohort study found that learning bimanual surgical skills in simulation with metric-specific instructions on AI-selected competencies was associated with unintended changes in other competency domains. These extrinsic outcomes had both positive and negative associations with learners’ expertise level compared with the skilled consultant’s benchmarks. Considering these unintended changes, assessment of AI-enhanced curricula requires a cost-benefit evaluation by subject matter experts to determine their acceptance in formal training. Finally, this AI-enhanced curriculum may benefit from contextual and timely feedback either from a live instructor or a real-time performance assessment system.

## Notes

Supplement 1. **eTable 1.** All Metrics Showing Significant Change During Entire Procedure (Condition 1) **eTable 2.** All Metrics Showing Significant Change During Tumor Removal (Condition 2)

Supplement 2.
Data Sharing Statement

## References

[1] Stulberg JJ, Huang R, Kreutzer L, . Association between surgeon technical skills and patient outcomes. JAMA Surg. 2020;155(10):960-968. doi:10.1001/jamasurg.2020.3007 32838425 PMC7439214

[2] Birkmeyer JD, Finks JF, O’Reilly A, ; Michigan Bariatric Surgery Collaborative. Surgical skill and complication rates after bariatric surgery. N Engl J Med. 2013;369(15):1434-1442. doi:10.1056/NEJMsa1300625 24106936

[3] Winkler-Schwartz A, Yilmaz R, Mirchi N, . Machine learning identification of surgical and operative factors associated with surgical expertise in virtual reality simulation. JAMA Netw Open. 2019;2(8):e198363. doi:10.1001/jamanetworkopen.2019.8363 31373651

[4] Mirchi N, Bissonnette V, Yilmaz R, Ledwos N, Winkler-Schwartz A, Del Maestro RF. The virtual operative assistant: an explainable artificial intelligence tool for simulation-based training in surgery and medicine. PLoS One. 2020;15(2):e0229596. doi:10.1371/journal.pone.0229596 32106247 PMC7046231

[5] Yilmaz R, Winkler-Schwartz A, Mirchi N, . Continuous monitoring of surgical bimanual expertise using deep neural networks in virtual reality simulation. NPJ Digit Med. 2022;5(1):54. doi:10.1038/s41746-022-00596-8 35473961 PMC9042967

[6] Mousavinasab E, Zarifsanaiey N. Niakan Kalhori SR, Rakhshan M, Keikha L, Saeedi MG. Intelligent tutoring systems: a systematic review of characteristics, applications, and evaluation methods. Interact Learn Environ. 2021;29(1):142-163. doi:10.1080/10494820.2018.1558257

[7] Mirchi N, Ledwos N, Del Maestro RF. Intelligent Tutoring Systems: Re-Envisioning Surgical Education in Response to COVID-19. Can J Neurol Sci. 2021;48(2):198-200. doi:10.1017/cjn.2020.202 32907644 PMC7642506

[8] Aggarwal R. Simulation in Surgical Education. In: Nestel D, Dalrymple K, Paige JT, Aggarwal R, eds. Advancing Surgical Education: Theory, Evidence and Practice. Springer Singapore; 2019:269-278. doi:10.1007/978-981-13-3128-2_24

[9] Dean WH, Gichuhi S, Buchan JC, . Intense simulation-based surgical education for manual small-incision cataract surgery: the Ophthalmic Learning and Improvement Initiative in Cataract Surgery randomized clinical trial in Kenya, Tanzania, Uganda, and Zimbabwe. JAMA Ophthalmol. 2021;139(1):9-15. doi:10.1001/jamaophthalmol.2020.4718 33151321 PMC7645744

[10] Meling TR, Meling TR. The impact of surgical simulation on patient outcomes: a systematic review and meta-analysis. Neurosurg Rev. 2021;44(2):843-854. doi:10.1007/s10143-020-01314-2 32399730 PMC8035110

[11] Frank JR, Mungroo R, Ahmad Y, Wang M, De Rossi S, Horsley T. Toward a definition of competency-based education in medicine: a systematic review of published definitions. Med Teach. 2010;32(8):631-637. doi:10.3109/0142159X.2010.500898 20662573

[12] Natheir S, Christie S, Yilmaz R, . Utilizing artificial intelligence and electroencephalography to assess expertise on a simulated neurosurgical task. Comput Biol Med. 2023;152:106286. doi:10.1016/j.compbiomed.2022.106286 36502696

[13] Effectiveness of an artificial intelligent tutoring system in simulation training. Accessed March 3, 2023. https://clinicaltrials.gov/ct2/show/NCT04700384

[14] Fazlollahi AM, Bakhaidar M, Alsayegh A, . Effect of artificial intelligence tutoring vs expert instruction on learning simulated surgical skills among medical students: a randomized clinical trial. JAMA Netw Open. 2022;5(2):e2149008. doi:10.1001/jamanetworkopen.2021.49008 35191972 PMC8864513

[15] Winkler-Schwartz A, Bissonnette V, Mirchi N, . Artificial intelligence in medical education: best practices using machine learning to assess surgical expertise in virtual reality simulation. J Surg Educ. 2019;76(6):1681-1690. doi:10.1016/j.jsurg.2019.05.015 31202633

[16] Sabbagh AJ, Bajunaid KM, Alarifi N, . Roadmap for developing complex virtual reality simulation scenarios: subpial neurosurgical tumor resection model. World Neurosurg. 2020;139:e220-e229. doi:10.1016/j.wneu.2020.03.187 32289510

[17] Gélinas-Phaneuf N, Choudhury N, Al-Habib AR, . Assessing performance in brain tumor resection using a novel virtual reality simulator. Int J Comput Assist Radiol Surg. 2014;9(1):1-9. doi:10.1007/s11548-013-0905-8 23784222

[18] Brunner WC, Korndorffer JR Jr, Sierra R, . Determining standards for laparoscopic proficiency using virtual reality. Am Surg. 2005;71(1):29-35. doi:10.1177/000313480507100105 15757053

[19] AlZhrani G, Alotaibi F, Azarnoush H, . Proficiency performance benchmarks for removal of simulated brain tumors using a virtual reality simulator NeuroTouch. J Surg Educ. 2015;72(4):685-696. doi:10.1016/j.jsurg.2014.12.014 25687956

[20] Althouse AD. Adjust for multiple comparisons: it’s not that simple. Ann Thorac Surg. 2016;101(5):1644-1645. doi:10.1016/j.athoracsur.2015.11.024 27106412

[21] Rothman KJ. No adjustments are needed for multiple comparisons. Epidemiology. 1990;1(1):43-46. doi:10.1097/00001648-199001000-00010 2081237

[22] Sawaya R, Alsideiri G, Bugdadi A, . Development of a performance model for virtual reality tumor resections. J Neurosurg. 2018;131(1):192-200. doi:10.3171/2018.2.JNS172327 30074456

[23] Swanwick T. Informal learning in postgraduate medical education: from cognitivism to ‘culturism’. Med Educ. 2005;39(8):859-865. doi:10.1111/j.1365-2929.2005.02224.x 16048629

[24] Schrewe B, Ellaway RH, Watling C, Bates J. The Contextual Curriculum: Learning in the Matrix, Learning From the Matrix. Acad Med. 2018;93(11):1645-1651. doi:10.1097/ACM.0000000000002345 29979208

[25] Yilmaz R, Ledwos N, Sawaya R, . Nondominant Hand Skills Spatial and Psychomotor Analysis During a Complex Virtual Reality Neurosurgical Task-A Case Series Study. Oper Neurosurg (Hagerstown). 2022;23(1):22-30. doi:10.1227/ons.0000000000000232 35726926

[26] Raghu Prasad MS, Manivannan M. Comparison of force matching performance in conventional and laparoscopic force-based task. Proc Hum Factors Ergon Soc Annu Meet. 2014;58(1):683-687. doi:10.1177/1541931214581160

[27] Nieboer TE, Sari V, Kluivers KB, Weinans MJ, Vierhout ME, Stegeman DF. A randomized trial of training the non-dominant upper extremity to enhance laparoscopic performance. Minim Invasive Ther Allied Technol. 2012;21(4):259-264. doi:10.3109/13645706.2011.614256 21939399

[28] Lee SH, Jin SH, An J. The difference in cortical activation pattern for complex motor skills: a functional near-infrared spectroscopy study. Sci Rep. 2019;9(1):14066. doi:10.1038/s41598-019-50644-9 31575954 PMC6773684

[29] Bravi R, Cohen EJ, Martinelli A, Gottard A, Minciacchi D. When non-dominant is better than dominant: kinesiotape modulates asymmetries in timed performance during a synchronization-continuation task. Front Integr Neurosci. 2017;11:21-21. doi:10.3389/fnint.2017.00021 28943842 PMC5596084

[30] Eppler MB, Sayegh AS, Maas M, . Automated capture of intraoperative adverse events using artificial intelligence: a systematic review and meta-analysis. J Clin Med. 2023;12(4):1687. doi:10.3390/jcm12041687 36836223 PMC9963108

[31] Levin M, McKechnie T, Kruse CC, Aldrich K, Grantcharov TP, Langerman A. Surgical data recording in the operating room: a systematic review of modalities and metrics. Br J Surg. 2021;108(6):613-621. doi:10.1093/bjs/znab016 34157080

[32] Khalid S, Goldenberg M, Grantcharov T, Taati B, Rudzicz F. Evaluation of deep learning models for identifying surgical actions and measuring performance. JAMA Netw Open. 2020;3(3):e201664. doi:10.1001/jamanetworkopen.2020.1664 32227178 PMC12124734

[33] Madani A, Namazi B, Altieri MS, . Artificial intelligence for intraoperative guidance: using semantic segmentation to identify surgical anatomy during laparoscopic cholecystectomy. Ann Surg. 2022;276(2):363-369. doi:10.1097/SLA.0000000000004594 33196488 PMC8186165

[34] Winkler-Schwartz A, Yilmaz R, Tran DH, . Creating a comprehensive research platform for surgical technique and operative outcome in primary brain tumor neurosurgery. World Neurosurg. 2020;144:e62-e71. doi:10.1016/j.wneu.2020.07.209 32758649

[35] Alsayegh A, Bakhaidar M, Winkler-Schwartz A, Yilmaz R, Del Maestro RF. Best practices using ex vivo animal brain models in neurosurgical education to assess surgical expertise. World Neurosurg. 2021;155:e369-e381. doi:10.1016/j.wneu.2021.08.061 34419656

[36] Hamdan NA. *Continuous Assessment of Instrument Tracking in an Ex Vivo Calf Brain Epilepsy Simulation Model*. Thesis. McGill University; 2023. Accessed July 5, 2023. https://escholarship.mcgill.ca/concern/theses/c534ft985

[37] Gordon L, Grantcharov T, Rudzicz F. Explainable artificial intelligence for safe intraoperative decision support. JAMA Surg. 2019;154(11):1064-1065. doi:10.1001/jamasurg.2019.2821 31509185

[38] Hebb AO, Yang T, Silbergeld DL. The sub-pial resection technique for intrinsic tumor surgery. Surg Neurol Int. 2011;2:180. doi:10.4103/2152-7806.90714 22368786 PMC3267372

[39] Siyar S, Azarnoush H, Rashidi S, . Machine learning distinguishes neurosurgical skill levels in a virtual reality tumor resection task. Med Biol Eng Comput. 2020;58(6):1357-1367. doi:10.1007/s11517-020-02155-3 32279203

[40] Alkadri S, Ledwos N, Mirchi N, . Utilizing a multilayer perceptron artificial neural network to assess a virtual reality surgical procedure. Comput Biol Med. 2021;136:104770. doi:10.1016/j.compbiomed.2021.104770 34426170

[41] Mirchi N, Bissonnette V, Ledwos N, . Artificial neural networks to assess virtual reality anterior cervical discectomy performance. Oper Neurosurg (Hagerstown). 2020;19(1):65-75. doi:10.1093/ons/opz359 31832652

[42] Reich A, Mirchi N, Yilmaz R, . Artificial neural network approach to competency-based training using a virtual reality neurosurgical simulation. Oper Neurosurg (Hagerstown). 2022;23(1):31-39. doi:10.1227/ons.0000000000000173 35726927

[43] Dihoff RE, Brosvic GM, Epstein ML, Cook MJ. Provision of feedback during preparation for academic testing: learning is enhanced by immediate but not delayed feedback. Psychol Rec. 2004;54(2):207-231. doi:10.1007/BF03395471

[44] Anderson DI, Magill RA, Sekiya H. Motor learning as a function of KR schedule and characteristics of task-intrinsic feedback. J Mot Behav. 2001;33(1):59-66. doi:10.1080/00222890109601903 11265058

[45] Lee JY, Szulewski A, Young JQ, Donkers J, Jarodzka H, van Merriënboer JJG. The medical pause: importance, processes and training. Med Educ. 2021;55(10):1152-1160. doi:10.1111/medu.14529 33772840 PMC8518691

[46] Yilmaz R, Alsayegh A, Bakhaidar M, . Comparing the efficacy of a real-time intelligent coaching system to human expert instruction in surgical technical skills training: randomized controlled trial. Neurooncol Adv. 2023;5(suppl 2):i1. doi:10.1093/noajnl/vdad071.00337287579

[47] Testing the efficacy of an artificial intelligence real-time coaching systemsystemsimulatiotraining of medical students. Accessed March 13, 2023. https://clinicaltrials.gov/ct2/show/NCT05168150

