# Supplementary material for: AI in Surgical Curriculum Design and Unintended Outcomes for Technical Competencies in Simulation Training
Source: JAMA Netw Open. 2023 Sep 19;6(9):e2334658. doi: 10.1001/jamanetworkopen.2023.34658 (PMC10509729; doi:10.1001/jamanetworkopen.2023.34658)
Supplement: Supplement 1. — eTable 1. All Metrics Showing Significant Change During Entire Procedure (Condition 1) eTable 2. All Metrics Showing Significant Change During Tumor Removal (Condition 2) [file jamanetwopen-e2334658-s001.pdf]

Supplemental Online Content

Fazlollahi AM, Yilmaz R, Winkler-Schwartz A, et al. AI in surgical curriculum design and unintended outcomes for technical competencies. *JAMA Netw Open*. 2023;6(9):e2334658. doi:10.1001/jamanetworkopen.2023.34658

eTable 1. All Metrics Showing Significant Change During Entire Procedure (Condition 1)
eTable 2. All Metrics Showing Significant Change During Tumor Removal (Condition 2)

This supplemental material has been provided by the authors to give readers additional information about their work.

© 2023 Fazlollahi AM et al. *JAMA Network Open.*

eTable 1. All Metrics Showing Significant Change During Entire Procedure (Condition 1)

Condition 1: Performance Metrics During the Entire Procedure							Within Group Comparison			Baseline Between Group Comparison			Post-Training Between Group Comparison			Expert Benchmarks (n=14)	
Metric	Group	Time	N	Mean	95.0% Lower CL for Mean	95.0% Upper CL for Mean	Mean Difference	Std. Error Difference	Sig.	Mean Difference	Std. Error Difference	Sig.	Mean Difference	Std. Error Difference	Sig.	Mean	Std. Deviation
maxTipDistance3D	Control	Baseline	23	8.32E+01	6.61E+01	1.00E+02	1.67E+00	1.07E+01	0.877	-1.48E+01	1.05E+01	0.166	-4.93E+01	8.06E+00	<.001	**6.19E+01**	**2.58E+01**
		Post-Training	23	8.49E+01	7.08E+01	9.90E+01											
	VOA	Baseline	23	6.84E+01	5.55E+01	8.13E+01	-3.28E+01	7.38E+00	<.001								
		Post-Training	23	3.56E+01	2.74E+01	4.38E+01											
meanConForceBipolarHand	Control	Baseline	23	6.58E-03	5.21E-03	7.95E-03	-6.71E-04	7.85E-04	0.397	-7.62E-04	7.69E-04	0.327	-2.61E-03	4.91E-04	<.001	**5.52E-03**	**1.28E-03**
		Post-Training	23	5.91E-03	5.04E-03	6.77E-03											
	VOA	Baseline	23	5.82E-03	5.09E-03	6.54E-03	-2.52E-03	4.20E-04	<.001								
		Post-Training	23	3.30E-03	2.82E-03	3.78E-03											
meanVelocity3DCusa	Control	Baseline	23	2.28E-01	1.95E-01	2.60E-01	3.89E-02	2.38E-02	0.109	-6.49E-03	2.35E-02	0.784	-1.32E-01	2.09E-02	<.001	**2.74E-01**	**3.73E-02**
		Post-Training	23	2.66E-01	2.30E-01	3.03E-01											
	VOA	Baseline	23	2.21E-01	1.85E-01	2.58E-01	-8.70E-02	2.01E-02	<.001								
		Post-Training	23	1.34E-01	1.14E-01	1.54E-01											
meanVelocity3DBipolar	Control	Baseline	23	2.13E-01	1.70E-01	2.57E-01	-2.33E-02	2.79E-02	0.409	-2.84E-02	2.69E-02	0.298	-9.68E-02	2.20E-02	<.001	**1.60E-01**	**4.15E-02**
		Post-Training	23	1.90E-01	1.52E-01	2.28E-01											
	VOA	Baseline	23	1.85E-01	1.51E-01	2.19E-01	-9.17E-02	1.98E-02	<.001								
		Post-Training	23	9.33E-02	7.04E-02	1.16E-01											
meanConTipDistance3D	Control	Baseline	23	1.86E-01	1.57E-01	2.14E-01	1.20E-02	2.17E-02	0.582	-1.90E-02	1.99E-02	0.343	-1.12E-01	1.93E-02	<.001	**1.73E-01**	**2.63E-02**
		Post-Training	23	1.98E-01	1.63E-01	2.32E-01											
	VOA	Baseline	23	1.67E-01	1.37E-01	1.97E-01	-8.06E-02	1.67E-02	<.001								
		Post-Training	23	8.62E-02	6.86E-02	1.04E-01											
meanConverging3D	Control	Baseline	23	1.89E-01	1.60E-01	2.19E-01	1.20E-02	2.18E-02	0.584	-1.83E-02	2.03E-02	0.373	-1.14E-01	1.88E-02	<.001	**1.77E-01**	**2.60E-02**
		Post-Training	23	2.01E-01	1.67E-01	2.35E-01											
	VOA	Baseline	23	1.71E-01	1.41E-01	2.01E-01	-8.38E-02	1.65E-02	<.001								

© 2023 Fazlollahi AM et al. *JAMA Network Open.*

Condition 1: Performance Metrics During the Entire Procedure							Within Group Comparison			Baseline Between Group Comparison			Post-Training Between Group Comparison			Expert Benchmarks (n=14)	
**Metric**	**Group**	**Time**	**N**	**Mean**	**95.0% Lower CL for Mean**	**95.0% Upper CL for Mean**	**Mean Difference**	**Std. Error Difference**	**Sig.**	**Mean Difference**	**Std. Error Difference**	**Sig.**	**Mean Difference**	**Std. Error Difference**	**Sig.**	**Mean**	**Std. Deviation**
		Post-Training	23	8.72E-02	7.01E-02	1.04E-01											
meanDiverging3D	Control	Baseline	23	1.83E-01	1.55E-01	2.10E-01	1.27E-02	2.20E-02	0.567	-1.96E-02	1.96E-02	0.323	-1.10E-01	2.01E-02	<.001	**1.69E-01**	**2.75E-02**
		Post-Training	23	1.95E-01	1.59E-01	2.32E-01											
	VOA	Baseline	23	1.63E-01	1.33E-01	1.93E-01	-7.78E-02	1.70E-02	<.001								
		Post-Training	23	8.53E-02	6.70E-02	1.04E-01											
meanVolume_tissueDr	Control	Baseline	23	-8.14E-05	-1.07E-04	-5.61E-05	-4.64E-05	2.04E-05	0.027	-1.41E-05	1.93E-05	0.468	7.05E-05	1.97E-05	<.001	**-9.26E-05**	**3.35E-05**
		Post-Training	23	-1.28E-04	-1.61E-04	-9.41E-05											
	VOA	Baseline	23	-9.55E-05	-1.27E-04	-6.41E-05	3.82E-05	1.83E-05	0.043								
		Post-Training	23	-5.74E-05	-7.88E-05	-3.59E-05											
maxVelocity3DCusa	Control	Baseline	23	4.79E+00	4.19E+00	5.39E+00	5.08E+00	5.26E-02	0.797	3.04E+00	4.81E-02	0.063	-6.46E+00	3.85E-02	<.001	**3.78E-01**	**8.85E-02**
		Post-Training	23	9.87E+00	4.13E+00	1.56E+01											
	VOA	Baseline	23	7.83E+00	2.57E+00	1.31E+01	-4.43E+00	2.92E-02	<.001								
		Post-Training	23	3.41E+00	1.87E+00	4.94E+00											
maxConForceBipolarHand	Control	Baseline	23	3.91E-01	3.10E-01	4.72E-01	-1.36E-02	3.83E-01	0.395	-9.18E-02	4.38E-01	0.421	-2.09E-01	3.50E-01	<.001	**4.98E+00**	**1.37E+00**
		Post-Training	23	3.77E-01	3.06E-01	4.49E-01											
	VOA	Baseline	23	2.99E-01	2.45E-01	3.53E-01	-1.31E-01	4.11E-01	<.001								
		Post-Training	23	1.68E-01	1.41E-01	1.96E-01											
maxConTipDistance3D	Control	Baseline	23	5.45E+00	4.92E+00	5.98E+00	2.77E+00	1.67E+00	0.104	7.44E-01	1.13E+00	0.512	-5.23E+00	1.86E+00	0.007	**5.75E+00**	**3.17E+00**
		Post-Training	23	8.22E+00	4.81E+00	1.16E+01											
	VOA	Baseline	23	6.19E+00	3.83E+00	8.56E+00	-3.21E+00	1.35E+00	0.022								
		Post-Training	23	2.98E+00	1.50E+00	4.47E+00											
maxDiverging3D	Control	Baseline	23	5.02E+00	4.54E+00	5.51E+00	2.82E+00	1.58E+00	0.080	1.08E+00	1.13E+00	0.341	-4.99E+00	1.74E+00	0.006	**5.14E+00**	**2.57E+00**
		Post-Training	23	7.85E+00	4.62E+00	1.11E+01											
	VOA	Baseline	23	6.11E+00	3.73E+00	8.49E+00	-3.25E+00	1.31E+00	0.017								
		Post-Training	23	2.86E+00	1.56E+00	4.16E+00											
	Control	Baseline	23	3.36E-03	2.29E-03	4.44E-03	-9.01E-04	5.50E-04	0.108	-5.84E-04	5.80E-04	0.319	-6.87E-04	2.15E-04	0.003	**2.72E-03**	**7.00E-04**

© 2023 Fazlollahi AM et al. *JAMA Network Open.*

**Condition 1: Performance Metrics During the Entire Procedure**							**Within Group Comparison**			**Baseline Between Group Comparison**			**Post-Training Between Group Comparison**			**Expert Benchmarks (n=14)**	
**Metric**	**Group**	**Time**	**N**	**Mean**	**95.0% Lower CL for Mean**	**95.0% Upper CL for Mean**	**Mean Difference**	**Std. Error Difference**	**Sig.**	**Mean Difference**	**Std. Error Difference**	**Sig.**	**Mean Difference**	**Std. Error Difference**	**Sig.**	**Mean**	**Std. Deviation**
medianConForceBipolarHand		Post-Training	23	2.46E-03	2.11E-03	2.82E-03											
	VOA	Baseline	23	2.78E-03	2.35E-03	3.21E-03	-1.00E-03	2.39E-04	<.001								
		Post-Training	23	1.78E-03	1.53E-03	2.03E-03											
medianConTipDistance3D	Control	Baseline	23	7.97E-02	6.77E-02	9.18E-02	4.55E-03	8.42E-03	0.592	-8.86E-04	8.79E-03	0.92	-3.58E-02	7.46E-03	<.001	**9.49E-02**	**1.30E-02**
		Post-Training	23	8.43E-02	7.18E-02	9.68E-02											
	VOA	Baseline	23	7.89E-02	6.52E-02	9.25E-02	-3.04E-02	7.79E-03	<.001								
		Post-Training	23	4.85E-02	3.99E-02	5.71E-02											
medianConverging3D	Control	Baseline	23	8.32E-02	7.03E-02	9.60E-02	5.99E-03	8.78E-03	0.498	-4.16E-05	9.55E-03	0.997	-3.98E-02	7.68E-03	<.001	**9.98E-02**	**1.44E-02**
		Post-Training	23	8.92E-02	7.64E-02	1.02E-01											
	VOA	Baseline	23	8.31E-02	6.79E-02	9.83E-02	-3.38E-02	8.51E-03	<.001								
		Post-Training	23	4.94E-02	4.04E-02	5.83E-02											
medianDiverging3D	Control	Baseline	23	7.66E-02	6.50E-02	8.83E-02	3.81E-03	8.17E-03	0.643	-9.69E-04	8.25E-03	0.907	-3.26E-02	7.27E-03	<.001	**9.12E-02**	**1.24E-02**
		Post-Training	23	8.04E-02	6.82E-02	9.26E-02											
	VOA	Baseline	23	7.57E-02	6.31E-02	8.82E-02	-2.78E-02	7.24E-03	<.001								
		Post-Training	23	4.79E-02	3.96E-02	5.62E-02											
meanAcceleration3DCusa	Control	Baseline	23	4.41E-02	3.84E-02	4.98E-02	5.27E-03	4.99E-03	0.296	-3.71E-04	4.37E-03	0.933	-2.25E-02	4.71E-03	<.001	**6.36E-02**	**1.02E-02**
		Post-Training	23	4.94E-02	4.08E-02	5.80E-02											
	VOA	Baseline	23	4.38E-02	3.67E-02	5.09E-02	-1.69E-02	3.93E-03	<.001								
		Post-Training	23	2.69E-02	2.29E-02	3.08E-02											
meanSpeeding3DCusa	Control	Baseline	23	4.46E-02	3.90E-02	5.03E-02	5.23E-03	4.93E-03	0.294	-2.67E-04	4.34E-03	0.951	-2.24E-02	4.68E-03	<.001	**6.50E-02**	**1.00E-02**
		Post-Training	23	4.99E-02	4.14E-02	5.84E-02											
	VOA	Baseline	23	4.44E-02	3.73E-02	5.15E-02	-1.69E-02	3.94E-03	<.001								
		Post-Training	23	2.75E-02	2.35E-02	3.16E-02											
meanSlowing3DCusa	Control	Baseline	23	4.35E-02	3.78E-02	4.92E-02	5.10E-03	4.90E-03	0.303	-4.28E-04	4.36E-03	0.922	-2.24E-02	4.58E-03	<.001	**6.20E-02**	**1.02E-02**
		Post-Training	23	4.86E-02	4.03E-02	5.69E-02											
	VOA	Baseline	23	4.31E-02	3.60E-02	5.01E-02	-1.69E-02	3.88E-03	<.001								

© 2023 Fazlollahi AM et al. *JAMA Network Open.*

Condition 1: Performance Metrics During the Entire Procedure							Within Group Comparison			Baseline Between Group Comparison			Post-Training Between Group Comparison			Expert Benchmarks (n=14)	
**Metric**	**Group**	**Time**	**N**	**Mean**	**95.0% Lower CL for Mean**	**95.0% Upper CL for Mean**	**Mean Difference**	**Std. Error Difference**	**Sig.**	**Mean Difference**	**Std. Error Difference**	**Sig.**	**Mean Difference**	**Std. Error Difference**	**Sig.**	**Mean**	**Std. Deviation**
		Post-Training	23	2.62E-02	2.23E-02	3.01E-02											
meanSlowing3DBipolar	Control	Baseline	23	4.39E-02	3.60E-02	5.18E-02	-9.08E-03	5.05E-03	0.078	-6.37E-03	4.89E-03	0.2	-1.47E-02	4.03E-03	<.001	**3.87E-02**	**1.08E-02**
		Post-Training	23	3.48E-02	2.81E-02	4.16E-02											
	VOA	Baseline	23	3.76E-02	3.14E-02	4.37E-02	-1.74E-02	3.69E-03	<.001								
		Post-Training	23	2.02E-02	1.56E-02	2.47E-02											
maxAcceleration3DBipolar	Control	Baseline	23	2.10E+00	1.77E+00	2.43E+00	6.33E-02	2.18E-01	0.773	-1.59E-01	2.26E-01	0.486	-1.24E+00	1.72E-01	<.001	**1.37E+00**	**5.43E-01**
		Post-Training	23	2.17E+00	1.86E+00	2.47E+00											
	VOA	Baseline	23	1.94E+00	1.62E+00	2.27E+00	-1.01E+00	1.77E-01	<.001								
		Post-Training	23	9.30E-01	7.66E-01	1.09E+00											
maxSpeeding3DBipolar	Control	Baseline	23	1.85E+00	1.60E+00	2.10E+00	6.52E-02	1.91E-01	0.734	-1.58E-01	1.91E-01	0.412	-1.03E+00	1.72E-01	<.001	**1.59E+00**	**5.08E-01**
		Post-Training	23	1.91E+00	1.61E+00	2.22E+00											
	VOA	Baseline	23	1.69E+00	1.38E+00	2.00E+00	-8.04E-01	1.70E-01	<.001								
		Post-Training	23	8.84E-01	7.15E-01	1.05E+00											
maxSlowing3DBipolar	Control	Baseline	23	1.98E+00	1.61E+00	2.35E+00	1.37E-02	2.27E-01	0.952	-1.62E-01	2.43E-01	0.51	-1.25E+00	1.57E-01	<.001	**1.72E+00**	**7.68E-01**
		Post-Training	23	1.99E+00	1.71E+00	2.28E+00											
	VOA	Baseline	23	1.82E+00	1.48E+00	2.15E+00	-1.07E+00	1.74E-01	<.001								
		Post-Training	23	7.50E-01	6.14E-01	8.85E-01											
medianAcceleration3DCusa	Control	Baseline	23	1.92E-02	1.71E-02	2.13E-02	1.24E-04	1.34E-03	0.927	-1.44E-04	1.58E-03	0.928	-5.85E-03	1.23E-03	<.001	**3.51E-02**	**4.10E-03**
		Post-Training	23	1.93E-02	1.75E-02	2.11E-02											
	VOA	Baseline	23	1.91E-02	1.65E-02	2.16E-02	-5.58E-03	1.50E-03	<.001								
		Post-Training	23	1.35E-02	1.17E-02	1.53E-02											
medianSlowing3DCusa	Control	Baseline	23	1.87E-02	1.67E-02	2.07E-02	2.89E-04	1.29E-03	0.824	-1.12E-04	1.53E-03	0.942	-5.66E-03	1.19E-03	<.001	**3.46E-02**	**4.25E-03**
		Post-Training	23	1.90E-02	1.73E-02	2.07E-02											
	VOA	Baseline	23	1.86E-02	1.61E-02	2.11E-02	-5.26E-03	1.45E-03	<.001								
		Post-Training	23	1.33E-02	1.16E-02	1.51E-02											
	Control	Baseline	23	4.93E-02	4.32E-02	5.54E-02	8.68E-03	6.22E-03	0.169	9.76E-04	5.23E-03	0.853	-2.56E-02	6.11E-03	<.001	**6.76E-02**	**1.38E-02**

© 2023 Fazlollahi AM et al. *JAMA Network Open.*

Condition 1: Performance Metrics During the Entire Procedure							Within Group Comparison			Baseline Between Group Comparison			Post-Training Between Group Comparison			Expert Benchmarks (n=14)	
**Metric**	**Group**	**Time**	**N**	**Mean**	**95.0% Lower CL for Mean**	**95.0% Upper CL for Mean**	**Mean Difference**	**Std. Error Difference**	**Sig.**	**Mean Difference**	**Std. Error Difference**	**Sig.**	**Mean Difference**	**Std. Error Difference**	**Sig.**	**Mean**	**Std. Deviation**
meanConAcceleration3DCusa		Post-Training	23	5.80E-02	4.67E-02	6.93E-02											
	VOA	Baseline	23	5.03E-02	4.12E-02	5.94E-02	-1.79E-02	4.96E-03	<.001								
		Post-Training	23	3.24E-02	2.76E-02	3.71E-02											
meanConAcceleration3DBipolar	Control	Baseline	23	5.09E-02	4.22E-02	5.96E-02	-1.04E-02	5.52E-03	0.066	-7.14E-03	5.41E-03	0.194	-1.53E-02	4.51E-03	0.001	**4.44E-02**	**1.30E-02**
		Post-Training	23	4.05E-02	3.31E-02	4.79E-02											
	VOA	Baseline	23	4.38E-02	3.69E-02	5.06E-02	-1.86E-02	4.23E-03	<.001								
		Post-Training	23	2.52E-02	1.97E-02	3.07E-02											
maxConAcceleration3DBipolar	Control	Baseline	23	2.73E+00	2.31E+00	3.15E+00	2.92E-01	3.17E-01	0.360	-2.66E-01	3.09E-01	0.393	-1.84E+00	2.68E-01	<.001	**2.34E+00**	**9.87E-01**
		Post-Training	23	3.02E+00	2.52E+00	3.53E+00											
	VOA	Baseline	23	2.47E+00	1.98E+00	2.95E+00	-1.28E+00	2.51E-01	<.001								
		Post-Training	23	1.18E+00	9.97E-01	1.37E+00											
medianConAcceleration3DCusa	Control	Baseline	23	2.18E-02	1.96E-02	2.40E-02	1.79E-03	1.44E-03	0.220	1.86E-04	1.70E-03	0.913	-6.71E-03	1.41E-03	<.001	**3.47E-02**	**4.15E-03**
		Post-Training	23	2.36E-02	2.16E-02	2.56E-02											
	VOA	Baseline	23	2.20E-02	1.92E-02	2.48E-02	-5.10E-03	1.69E-03	0.004								
		Post-Training	23	1.69E-02	1.48E-02	1.90E-02											
meanForceBipolarHand	Control	Baseline	23	3.64E-01	2.94E-01	4.33E-01	1.37E-01	4.82E-02	0.007	-4.26E-02	4.50E-02	0.348	-2.90E-01	4.21E-02	<.001	**4.06E-01**	**8.68E-02**
		Post-Training	23	5.00E-01	4.29E-01	5.72E-01											
	VOA	Baseline	23	3.21E-01	2.60E-01	3.82E-01	-1.11E-01	3.71E-02	0.005								
		Post-Training	23	2.10E-01	1.63E-01	2.57E-01											
meanTipDistance3D	Control	Baseline	23	1.95E+01	1.47E+01	2.43E+01	-4.36E+00	2.82E+00	0.129	-1.07E+00	3.81E+00	0.78	-8.97E+00	1.69E+00	<.001	**7.85E+00**	**1.65E+00**
		Post-Training	23	1.52E+01	1.19E+01	1.85E+01											
	VOA	Baseline	23	1.85E+01	1.21E+01	2.48E+01	-1.23E+01	3.09E+00	<.001								
		Post-Training	23	6.21E+00	5.47E+00	6.94E+00											
maxForceBipolarHand	Control	Baseline	23	9.81E-01	8.44E-01	1.12E+00	1.56E-01	9.12E-02	0.094	-5.95E-03	9.37E-02	0.95	-5.16E-01	8.58E-02	<.001	**8.57E-01**	**1.47E-01**
		Post-Training	23	1.14E+00	1.01E+00	1.27E+00											
	VOA	Baseline	23	9.75E-01	8.38E-01	1.11E+00	-3.54E-01	8.79E-02	<.001								

© 2023 Fazlollahi AM et al. *JAMA Network Open.*

Condition 1: Performance Metrics During the Entire Procedure							Within Group Comparison			Baseline Between Group Comparison			Post-Training Between Group Comparison			Expert Benchmarks (n=14)	
**Metric**	**Group**	**Time**	**N**	**Mean**	**95.0% Lower CL for Mean**	**95.0% Upper CL for Mean**	**Mean Difference**	**Std. Error Difference**	**Sig.**	**Mean Difference**	**Std. Error Difference**	**Sig.**	**Mean Difference**	**Std. Error Difference**	**Sig.**	**Mean**	**Std. Deviation**
		Post-Training	23	6.21E-01	5.00E-01	7.41E-01											
medianForceBipolarHand	Control	Baseline	23	3.55E-01	2.72E-01	4.37E-01	1.54E-01	5.50E-02	0.007	-4.88E-02	5.43E-02	0.374	-3.11E-01	4.54E-02	<.001	**4.14E-01**	**1.06E-01**
		Post-Training	23	5.08E-01	4.30E-01	5.86E-01											
	VOA	Baseline	23	3.06E-01	2.31E-01	3.81E-01	-1.09E-01	4.32E-02	0.015								
		Post-Training	23	1.97E-01	1.48E-01	2.46E-01											
medianTipDistance3D	Control	Baseline	23	1.73E+01	1.24E+01	2.22E+01	-5.74E+00	2.85E+00	0.049	-1.55E+00	4.09E+00	0.707	-6.22E+00	1.67E+00	<.001	**6.22E+00**	**1.08E+00**
		Post-Training	23	1.15E+01	8.29E+00	1.48E+01											
	VOA	Baseline	23	1.57E+01	8.68E+00	2.28E+01	-1.04E+01	3.41E+00	0.004								
		Post-Training	23	5.33E+00	4.77E+00	5.88E+00											
medianVelocity3DCusa	Control	Baseline	23	1.18E-01	1.05E-01	1.31E-01	2.65E-02	8.61E-03	0.003	6.63E-03	1.09E-02	0.545	-5.03E-02	8.60E-03	<.001	**1.79E-01**	**2.31E-02**
		Post-Training	23	1.45E-01	1.33E-01	1.57E-01											
	VOA	Baseline	23	1.25E-01	1.06E-01	1.44E-01	-3.05E-02	1.10E-02	0.008								
		Post-Training	23	9.44E-02	8.15E-02	1.07E-01											
medianVelocity3DBipolar	Control	Baseline	23	8.19E-02	6.18E-02	1.02E-01	-2.58E-02	1.29E-02	0.051	-1.09E-02	1.25E-02	0.39	-7.92E-03	1.03E-02	0.445	**5.44E-02**	**1.70E-02**
		Post-Training	23	5.61E-02	3.88E-02	7.35E-02											
	VOA	Baseline	23	7.10E-02	5.53E-02	8.68E-02	-2.28E-02	9.44E-03	0.02								
		Post-Training	23	4.82E-02	3.66E-02	5.98E-02											
meanAcceleration3DBipolar	Control	Baseline	23	4.50E-02	3.71E-02	5.29E-02	-9.12E-03	5.08E-03	0.079	-6.29E-03	4.93E-03	0.209	-1.52E-02	4.10E-03	<.001	**3.96E-02**	**1.10E-02**
		Post-Training	23	3.59E-02	2.90E-02	4.28E-02											
	VOA	Baseline	23	3.87E-02	3.24E-02	4.50E-02	-1.80E-02	3.77E-03	<.001								
		Post-Training	23	2.07E-02	1.60E-02	2.53E-02											
meanSpeeding3DBipolar	Control	Baseline	23	4.60E-02	3.81E-02	5.39E-02	-9.12E-03	5.11E-03	0.081	-6.15E-03	4.96E-03	0.222	-1.57E-02	4.18E-03	<.001	**4.06E-02**	**1.12E-02**
		Post-Training	23	3.69E-02	2.99E-02	4.39E-02											
	VOA	Baseline	23	3.99E-02	3.35E-02	4.63E-02	-1.87E-02	3.86E-03	<.001								
		Post-Training	23	2.12E-02	1.64E-02	2.60E-02											
	Control	Baseline	23	1.37E-02	1.04E-02	1.69E-02	-6.27E-03	1.88E-03	0.002	-2.04E-03	1.94E-03	0.297	2.71E-04	1.52E-03	0.859	**1.12E-02**	**4.16E-03**

© 2023 Fazlollahi AM et al. *JAMA Network Open.*

Condition 1: Performance Metrics During the Entire Procedure							Within Group Comparison			Baseline Between Group Comparison			Post-Training Between Group Comparison			Expert Benchmarks (n=14)	
**Metric**	**Group**	**Time**	**N**	**Mean**	**95.0% Lower CL for Mean**	**95.0% Upper CL for Mean**	**Mean Difference**	**Std. Error Difference**	**Sig.**	**Mean Difference**	**Std. Error Difference**	**Sig.**	**Mean Difference**	**Std. Error Difference**	**Sig.**	**Mean**	**Std. Deviation**
medianAcceleration3DBipolar		Post-Training	23	7.42E-03	5.28E-03	9.57E-03											
	VOA	Baseline	23	1.17E-02	9.43E-03	1.39E-02	-3.96E-03	1.55E-03	0.014								
		Post-Training	23	7.70E-03	5.38E-03	1.00E-02											
medianSpeeding3DBipolar	Control	Baseline	23	1.41E-02	1.08E-02	1.75E-02	-6.51E-03	1.95E-03	0.002	-1.99E-03	2.01E-03	0.327	1.92E-04	1.58E-03	0.904	**1.14E-02**	**4.27E-03**
		Post-Training	23	7.60E-03	5.36E-03	9.85E-03											
	VOA	Baseline	23	1.21E-02	9.79E-03	1.45E-02	-4.33E-03	1.61E-03	0.010								
		Post-Training	23	7.80E-03	5.41E-03	1.02E-02											
medianSlowing3DBipolar	Control	Baseline	23	1.33E-02	1.01E-02	1.64E-02	-6.02E-03	1.82E-03	0.002	-2.03E-03	1.87E-03	0.282	3.39E-04	1.47E-03	0.819	**1.11E-02**	**4.09E-03**
		Post-Training	23	7.25E-03	5.19E-03	9.31E-03											
	VOA	Baseline	23	1.12E-02	9.11E-03	1.34E-02	-3.64E-03	1.49E-03	0.019								
		Post-Training	23	7.59E-03	5.34E-03	9.84E-03											
medianConAcceleration3DBipolar	Control	Baseline	23	1.55E-02	1.20E-02	1.91E-02	-6.73E-03	1.99E-03	0.001	-2.04E-03	2.12E-03	0.341	8.85E-04	1.72E-03	0.609	**1.17E-02**	**4.71E-03**
		Post-Training	23	8.81E-03	6.73E-03	1.09E-02											
	VOA	Baseline	23	1.35E-02	1.10E-02	1.60E-02	-3.80E-03	1.85E-03	0.046								
		Post-Training	23	9.70E-03	6.77E-03	1.26E-02											

© 2023 Fazlollahi AM et al. *JAMA Network Open.*

Table 2. All Metrics Showing Significant Change During Tumor Removal (Condition 2)

Condition 2: Performance Metrics During Tumor Removal							Within Group Comparison			Baseline Between Group Comparison			Post-Training Between Group Comparison			Expert Benchmarks (n=14)	
**Metric**	**Group**	**Time**	**N**	**Mean**	**95.0% Lower CL for Mean**	**95.0% Upper CL for Mean**	**Mean Difference**	**Std. Error Difference**	**Sig.**	**Mean Difference**	**Std. Error Difference**	**Sig.**	**Mean Difference**	**Std. Error Difference**	**Sig.**	**Mean**	**Std. Deviation**
meanForceBipolarHand	Control	Baseline	23	4.92E-01	3.80E-01	6.04E-01	8.05E-02	6.86E-02	0.246	-9.02E-02	6.90E-02	0.198	-3.46E-01	5.05E-02	<.001	2.73E-01	7.02E-02
		Post-training	23	5.73E-01	4.86E-01	6.59E-01											
	VOA	Baseline	23	4.02E-01	3.17E-01	4.87E-01	-1.75E-01	4.87E-02	<.001								
		Post-training	23	2.27E-01	1.72E-01	2.81E-01											
meanTipDistance3D	Control	Baseline	23	1.58E+01	1.14E+01	2.01E+01	-2.85E+00	2.57E+00	0.272	3.66E+00	4.82E+00	0.451	-6.93E+00	1.53E+00	<.001	7.87E+00	2.38E+00
		Post-training	23	1.29E+01	9.93E+00	1.59E+01											
	VOA	Baseline	23	1.94E+01	1.02E+01	2.87E+01	-1.34E+01	4.47E+00	0.004								
		Post-training	23	5.98E+00	5.32E+00	6.63E+00											
maxTipDistance3D	Control	Baseline	23	3.81E+01	3.24E+01	4.38E+01	1.68E+00	5.03E+00	0.74	4.80E+00	6.23E+00	0.445	-2.41E+01	4.66E+00	<.001	3.41E+01	1.71E+01
		Post-training	23	3.98E+01	3.11E+01	4.84E+01											
	VOA	Baseline	23	4.29E+01	3.10E+01	5.48E+01	-2.72E+01	5.99E+00	<.001								
		Post-training	23	1.57E+01	1.23E+01	1.92E+01											
medianForceBipolarHand	Control	Baseline	23	4.86E-01	3.66E-01	6.05E-01	7.34E-02	7.12E-02	0.308	-9.48E-02	7.50E-02	0.212	-3.39E-01	5.08E-02	<.001	2.67E-01	8.28E-02
		Post-training	23	5.59E-01	4.74E-01	6.45E-01											
	VOA	Baseline	23	3.91E-01	2.95E-01	4.87E-01	-1.71E-01	5.40E-02	0.003								
		Post-training	23	2.20E-01	1.63E-01	2.78E-01											
meanConForceBipolarHand	Control	Baseline	23	5.07E-03	3.21E-03	6.93E-03	-1.23E-03	9.32E-04	0.195	-7.81E-04	1.01E-03	0.441	-8.98E-04	3.26E-04	0.008	1.08E-02	3.12E-03
		Post-training	23	3.85E-03	3.35E-03	4.34E-03											
	VOA	Baseline	23	4.29E-03	3.51E-03	5.07E-03	-1.34E-03	4.34E-04	0.003								
		Post-training	23	2.95E-03	2.50E-03	3.40E-03											
meanVelocity3DCusa	Control	Baseline	23	1.49E-01	1.25E-01	1.72E-01	1.46E-02	1.33E-02	0.28	3.95E-04	1.63E-02	0.981	-4.33E-02	1.07E-02	<.001	2.70E-01	8.33E-02
		Post-training	23	1.63E-01	1.49E-01	1.77E-01											
	VOA	Baseline	23	1.49E-01	1.25E-01	1.73E-01	-2.92E-02	1.43E-02	0.048								
		Post-training	23	1.20E-01	1.03E-01	1.37E-01											
meanConverging3D	Control	Baseline	23	8.43E-02	7.05E-02	9.80E-02	3.56E-03	7.83E-03	0.651	3.52E-03	1.01E-02	0.729	-2.25E-02	6.98E-03	0.002	1.92E-01	4.28E-02
		Post-training	23	8.78E-02	7.93E-02	9.63E-02											
	VOA	Baseline	23	8.78E-02	7.19E-02	1.04E-01	-2.24E-02	9.56E-03	0.024								
		Post-training	23	6.54E-02	5.34E-02	7.73E-02											
maxConForceBipolarHand	Control	Baseline	23	1.88E-01	1.21E-01	2.54E-01	1.14E-02	4.09E-02	0.782	-3.52E-02	3.92E-02	0.374	-1.11E-01	2.74E-02	<.001	2.36E-01	9.80E-02
		Post-training	23	1.99E-01	1.47E-01	2.51E-01											
	VOA	Baseline	23	1.52E-01	1.09E-01	1.96E-01	-6.40E-02	2.26E-02	0.007								

© 2023 Fazlollahi AM et al. *JAMA Network Open.*

Condition 2: Performance Metrics During Tumor Removal							Within Group Comparison			Baseline Between Group Comparison			Post-Training Between Group Comparison			Expert Benchmarks (n=14)	
**Metric**	**Group**	**Time**	**N**	**Mean**	**95.0% Lower CL for Mean**	**95.0% Upper CL for Mean**	**Mean Difference**	**Std. Error Difference**	**Sig.**	**Mean Difference**	**Std. Error Difference**	**Sig.**	**Mean Difference**	**Std. Error Difference**	**Sig.**	**Mean**	**Std. Deviation**
		Post-training	23	8.84E-02	7.15E-02	1.05E-01											
maxVelocity3DBipolar	Control	Baseline	23	1.71E+00	1.30E+00	2.12E+00	1.34E-01	2.76E-01	0.629	-7.83E-02	2.62E-01	0.766	-1.14E+00	2.16E-01	<.001	3.31E+00	1.28E+00
		Post-training	23	1.84E+00	1.44E+00	2.24E+00											
	VOA	Baseline	23	1.63E+00	1.28E+00	1.97E+00	-9.26E-01	1.86E-01	<.001								
		Post-training	23	7.01E-01	5.28E-01	8.74E-01											
maxDiverging3D	Control	Baseline	23	1.39E+00	9.72E-01	1.81E+00	3.14E-01	2.76E-01	0.262	4.43E-02	2.79E-01	0.875	-1.10E+00	2.09E-01	<.001	2.72E+00	1.35E+00
		Post-training	23	1.70E+00	1.32E+00	2.09E+00											
	VOA	Baseline	23	1.43E+00	1.04E+00	1.83E+00	-8.33E-01	2.05E-01	<.001								
		Post-training	23	6.02E-01	4.43E-01	7.62E-01											
meanAcceleration3DCusa	Control	Baseline	23	2.51E-02	2.21E-02	2.80E-02	2.41E-03	1.88E-03	0.205	3.74E-03	4.15E-03	0.372	-7.19E-03	1.81E-03	<.001	6.12E-02	1.52E-02
		Post-training	23	2.75E-02	2.50E-02	3.00E-02											
	VOA	Baseline	23	2.88E-02	2.05E-02	3.72E-02	-8.52E-03	4.26E-03	0.047								
		Post-training	23	2.03E-02	1.75E-02	2.31E-02											
meanSlowing3DCusa	Control	Baseline	23	2.49E-02	2.19E-02	2.79E-02	2.09E-03	1.85E-03	0.264	1.34E-03	2.58E-03	0.606	-5.97E-03	1.77E-03	0.001	5.54E-02	1.03E-02
		Post-training	23	2.70E-02	2.46E-02	2.93E-02											
	VOA	Baseline	23	2.62E-02	2.17E-02	3.07E-02	-5.23E-03	2.57E-03	0.048								
		Post-training	23	2.10E-02	1.82E-02	2.38E-02											
maxAcceleration3DBipolar	Control	Baseline	23	6.30E-01	4.68E-01	7.91E-01	-2.10E-02	1.08E-01	0.847	-1.16E-01	1.11E-01	0.298	-3.65E-01	8.24E-02	<.001	3.55E-01	1.31E-01
		Post-training	23	6.09E-01	4.54E-01	7.63E-01											
	VOA	Baseline	23	5.13E-01	3.52E-01	6.75E-01	-2.69E-01	8.24E-02	0.002								
		Post-training	23	2.44E-01	1.87E-01	3.01E-01											
maxSpeeding3DBipolar	Control	Baseline	23	5.62E-01	4.07E-01	7.17E-01	-2.48E-02	1.03E-01	0.811	-1.04E-01	1.07E-01	0.335	-3.20E-01	7.79E-02	<.001	9.55E-01	3.76E-01
		Post-training	23	5.37E-01	3.92E-01	6.82E-01											
	VOA	Baseline	23	4.58E-01	3.01E-01	6.15E-01	-2.41E-01	8.06E-02	0.005								
		Post-training	23	2.17E-01	1.59E-01	2.74E-01											
maxSlowing3DBipolar	Control	Baseline	23	3.89E-01	2.64E-01	5.13E-01	4.86E-02	9.47E-02	0.612	-2.61E-02	7.75E-02	0.737	-2.32E-01	7.89E-02	0.005	8.95E-01	3.48E-01
		Post-training	23	4.37E-01	2.87E-01	5.88E-01											
	VOA	Baseline	23	3.63E-01	2.65E-01	4.60E-01	-1.57E-01	5.17E-02	0.004								
		Post-training	23	2.05E-01	1.61E-01	2.49E-01											
medianSpeeding3DCusa	Control	Baseline	23	1.22E-02	1.08E-02	1.36E-02	2.88E-05	8.04E-04	0.972	-7.91E-05	1.12E-03	0.944	-2.58E-03	7.95E-04	0.002	3.76E-02	1.29E-02
		Post-training	23	1.22E-02	1.13E-02	1.31E-02											
	VOA	Baseline	23	1.21E-02	1.02E-02	1.39E-02	-2.48E-03	1.13E-03	0.034								

© 2023 Fazlollahi AM et al. *JAMA Network Open.*

Condition 2: Performance Metrics During Tumor Removal							Within Group Comparison			Baseline Between Group Comparison			Post-Training Between Group Comparison			Expert Benchmarks (n=14)	
**Metric**	**Group**	**Time**	**N**	**Mean**	**95.0% Lower CL for Mean**	**95.0% Upper CL for Mean**	**Mean Difference**	**Std. Error Difference**	**Sig.**	**Mean Difference**	**Std. Error Difference**	**Sig.**	**Mean Difference**	**Std. Error Difference**	**Sig.**	**Mean**	**Std. Deviation**
		Post-training	23	9.60E-03	8.19E-03	1.10E-02											
maxConAcceleration3DBipolar	Control	Baseline	23	6.90E-01	5.14E-01	8.65E-01	-2.88E-02	1.14E-01	0.802	-6.03E-02	1.16E-01	0.607	-3.28E-01	8.73E-02	<.001	1.28E+00	5.06E-01
		Post-training	23	6.61E-01	5.04E-01	8.18E-01											
	VOA	Baseline	23	6.30E-01	4.67E-01	7.93E-01	-2.97E-01	8.71E-02	0.001								
		Post-training	23	3.33E-01	2.55E-01	4.11E-01											
maxForceBipolarHand	Control	Baseline	23	8.84E-01	7.36E-01	1.03E+00	1.58E-01	9.20E-02	0.091	-9.09E-02	9.39E-02	0.338	-4.84E-01	8.22E-02	<.001	6.78E-01	1.33E-01
		Post-training	23	1.04E+00	9.24E-01	1.16E+00											
	VOA	Baseline	23	7.93E-01	6.71E-01	9.16E-01	-2.35E-01	8.32E-02	0.007								
		Post-training	23	5.59E-01	4.37E-01	6.80E-01											
medianTipDistance3D	Control	Baseline	23	1.42E+01	9.35E+00	1.91E+01	-4.41E+00	2.67E+00	0.105	4.10E+00	5.48E+00	0.458	-4.21E+00	1.34E+00	0.003	6.80E+00	2.38E+00
		Post-training	23	9.80E+00	7.20E+00	1.24E+01											
	VOA	Baseline	23	1.83E+01	7.70E+00	2.89E+01	-1.27E+01	5.12E+00	0.017								
		Post-training	23	5.59E+00	5.00E+00	6.19E+00											
meanDiverging3D	Control	Baseline	23	8.10E-02	6.85E-02	9.34E-02	1.03E-02	7.24E-03	0.161	3.37E-03	8.70E-03	0.7	-2.50E-02	6.64E-03	<.001	2.44E-01	7.93E-02
		Post-training	23	9.13E-02	8.31E-02	9.95E-02											
	VOA	Baseline	23	8.43E-02	7.14E-02	9.73E-02	-1.81E-02	8.27E-03	0.034								
		Post-training	23	6.62E-02	5.50E-02	7.74E-02											

© 2023 Fazlollahi AM et al. *JAMA Network Open.*
