# Supplementary material for: AI in Surgical Curriculum Design and Unintended Outcomes for Technical Competencies in Simulation Training
Source: JAMA Netw Open. 2023 Sep 19;6(9):e2334658. doi: 10.1001/jamanetworkopen.2023.34658 (PMC10509729; doi:10.1001/jamanetworkopen.2023.34658)
Supplement: Supplement 2. — Data Sharing Statement [file jamanetwopen-e2334658-s002.pdf]

Data Sharing Statement

Fazlollahi. AI in Surgical Curriculum Design and Unintended Outcomes for Technical Competencies in Simulation Training. *JAMA Netw Open*. Published September 19, 2023.
doi:10.1001/jamanetworkopen.2023.34658

Data
Data available: Yes
Data types: Deidentified participant data
How to access data: Upon request from authors Fazlollahi and Del Maestro. Contact ali.fazlollahi@mail.mcgill.ca and rolando.del_maestro@mcgill.ca.
When available: With publication

Supporting Documents
Document types: None

Additional Information
Who can access the data: Researchers whose proposed use of the data has been approved.
Types of analyses: Any purpose.
Mechanisms of data availability: With a signed data access agreement.
